# Major and minor ECG abnormalities depending on regional living conditions in Russia

**DOI:** 10.1038/s41598-023-35947-2

**Published:** 2023-06-01

**Authors:** Sergey Maksimov, Galina Muromtseva, Vladimir Kutsenko, Svetlana Shalnova, Svetlana Evstifeeva, Oksana Drapkina

**Affiliations:** National Medical Research Center for Therapy and Preventive Medicine, Moscow, Russian Federation

**Keywords:** Cardiology, Risk factors, Environmental impact, Ecological epidemiology

## Abstract

The goal of our study was to explore the effect of living conditions on the odd of major and minor ECG abnormalities on a large region scale in Russia. For the analysis, cross-sectional data of the Russian study, ESSE-RF 2013–2014, were used. They were collected on a sample of 16,400 subjects from 10 regions of the Russia. ECG abnormalities were grouped into two categories: Major and Minor (sensu the 2009 version of the Minnesota Code Classification System). Regional living conditions were considered comprehensively via five indices combining 33 characteristics of the regions. The estimates were presented as odds ratios and their 95% confidence intervals. The prevalence values of major abnormalities in the sample were 8.4% among women and 9.4% among men (p = 0.021). The prevalence of minor abnormalities constituted 34.1% and 45.9%, respectively (p < 0.001). In men, the odd of major ECG abnormalities increased with the demographic depression growth (1.08: 1.04–1.12) and with industrial development growth in the region (1.12: 1.07–1.17). In women, an increase in the odd of major ECG abnormalities was directly associated with industrial development (1.12: 1.07–1.16) and inversely related to the economic development in the region (0.94: 0.89–0.99). The odd of minor ECG abnormalities in men and women declined with the growth of the regional economic development: OR of 0.95: 0.93–0.98, and OR of 0.92: 0.87–0.99, respectively. The study demonstrated an effect of regional living conditions of the Russian population on the odd of major and minor ECG abnormalities. The most stable and logically explainable relationships were obtained for industrial and economic characteristics of living conditions.

## Introduction

Numerous published studies claimed high prospects of using the Minnesota Code Classification System of electrocardiograms (ECG) for diagnosing clinical forms of coronary artery disease (CAD) and heart failure, along with the prognosis of deaths from cardiovascular diseases^1–5^. The prevalence of major and minor ECG abnormalities was studied in the populations of different countries, including Belgium^6^, China^7^, Netherlands^8^, USA^9^, Pakistan^10^, Brazil^11^, Singapore^12^, Poland^13^, and Russia^14^. In addition to gender, age and race, some studies considered other conventional individual cardiovascular risk factors for the prevalence of ECG abnormalities in the population^8,9,11,15–17^. However, the prevalence patterns of ECG abnormalities, depending on environmental conditions, were not sufficiently studied at the population level. In fact, the relevant search among available literature yielded just two Chinese publications that considered the spatial aspects of the prevalence of ECG abnormalities^18,19^. It is worth noting that in these sources, spatial features were considered without taking into account the living conditions of the population; hence, the authors could only state geographical differences in prevalence without analyzing their causes.

Living conditions are currently considered among significant factors in the formation of individual health, including cardiovascular well-being^20–22^. Depending on living conditions and specific health indicators, the external environment can have both positive and unfavorable direct and/or indirect effects. In one of the first publications on the topic, Diez Roux AV et al. demonstrated the dependence of the CAD prevalence on the socioeconomic characteristics in the area of residence, including median household income, proportion of the adult population in the area with a secondary school education, etc.^23,24^. Subsequently, the effect of deprivation of residential area on the prevalence of CAD was confirmed in other publications as well^25–28^. Besides living conditions at the district level, an impact of income inequality (Gini index) on the risk of heart attack^29^, as well as of regional socioeconomic characteristics in childhood on the likelihood of CAD, at the level of large regions (e.g. states in the USA). At the same time, ECG abnormalities as a diagnostic tool for CAD and prediction of cardiovascular events were not considered from the standpoint of dependence on regional living conditions of the population, despite the fact that a large amount of scientific data were devoted to the influence of regional living conditions on other indicators of cardiovascular risk^30,31^. The goal of our study was to explore the effect of living conditions on the likelihood of major and minor ECG abnormalities on a large region scale in Russia.

## Methods

### Characterization of the sample

For the analysis, we used data from the cross-sectional epidemiological study, *Epidemiology of Cardiovascular Diseases in the Regions of the Russian Federation* (ESSE-RF), conducted in 2013–2014. A total of 21,923 subjects 25–64 years of age in 13 regions of the Russian Federation participated in the study. When forming the sample, the Kish grid method was employed, ensuring a systematic multistage random sampling based on the territorial principle (on the basis of medical institutions). The response rate to the survey was approximately 80%, ranging across study regions.

The study was approved by the Ethics Committees of National Research Center for Therapy and Preventive Medicine (Moscow, Russian Federation) No. 07-03/12 (03.07.2012). The study was performed in compliance with Good Clinical Practice and the Declaration of Helsinki principles. Written informed consent was obtained from all participants prior to their enrollment in the study.

ECG was performed in 11 regions. St. Petersburg was excluded from the final sample, because it differed significantly in its regional characteristics from ten other regions. St. Petersburg is classified in the Russian Federation as a separate administrative territorial unit, while ten other regions are large territories, including both cities and rural areas. The characteristics of 10 regions included in the study regarding cardiovascular morbidity and mortality are presented in Table 1. In 2010–2014, their values varied in a fairly wide range (cardiovascular mortality: from 494.6 to 902.7 cases per 100 thousand population in Tyumen Region and Vologda Region, correspondingly; cardiovascular morbidity: from 18.4 to 38.6 cases per 1000 population in Primorsky Krai and Kemerovo Region, respectively).

**Table 1:** Cardiovascular mortality and morbidity in the study regions in 2010–2014.

Region	Mortality rate from circulatory diseases, per 100,000 people						Incidence of circulatory system diseases per 1000 population (in patients with a diagnosis established for the first time in their lives)					
	2010	2011	2012	2013	2014	Mean 2010–2014	2010	2011	2012	2013	2014	Mean 2010–2014
Primorsky Krai	785.1	767.4	743.8	729.0	732.1	751.5	19.5	18.9	16.6	17.1	19.7	18.4
Republic of North Ossetia	697.8	682.4	689.8	684.1	687.6	688.3	25.4	24.5	31.8	38.3	35.3	31.1
Volgograd region	910.4	820.5	764.5	752.7	745.6	798.7	22.3	20.4	22.8	31.4	26.8	24.7
Vologda region	968.8	927.9	896.0	892.2	828.4	902.7	28.1	25.2	24.3	22.3	22.9	24.6
Voronezh region	1029.3	928.1	872.3	763.3	744.1	867.4	27.2	28.2	27.6	29.2	35.5	29.5
Ivanovo region	946.5	730.1	698.9	645.4	639.4	732.1	28.1	28.0	25.5	24.5	23.6	25.9
Kemerovo region	778.5	740.8	702.4	647.7	614.7	696.8	31.2	35.0	36.1	44.7	46.2	38.6
Krasnoyarsk region	659.3	609.3	614.5	610.6	596.5	618.0	33.2	33.1	32.6	34.4	32.9	33.2
Tomsk region	612.9	573.9	528.6	519.6	515.5	550.1	17.0	23.6	21.1	21.5	19.8	20.6
Tyumen region	436.5	423.6	414.1	599.6	599.1	494.6	22.5	22.9	25.3	25.9	24.4	24.2

For some individual indicators considered as covariates, there were missing or incomplete data: marital status (n = 126 or 0.8% of the final sample), education (n = 15 or 0.1%), employment (n = 12 or 0.1%), income (n = 264 or 1.6%), hypertension (n = 441 or 2.7%), obesity (n = 203 or 1.2%), hypercholesterolemia (n = 633 or 3.9%), diabetes mellitus (n = 738 or 4.5%), dietary pattern (n = 1876 or 11.4%), alcohol consumption (n = 1774 or 10.8%), and smoking (n = 28 or 0.2%). For these indicators, the missing data was restored using the k-nearest neighbors’ algorithm according to the following input parameters: region, place of residence (urban/rural), gender, and age. Hence, the final sample included 16,400 subjects representing 10 regions, comprising 6305 men and 10,095 women.

### Major and minor ECG abnormalities

Recording of 12 ECG leads at rest was carried out according to the same protocol at a medical institution, in the supine position after a 5-min rest, on a PADSY computer ECG complex (Medset Medizintechnik GmbH, Hamburg, Germany). ECG recordings from the regions were sent electronically to the National Medical Research Center for Therapy and Preventive Medicine of the Russian Federation Ministry of Healthcare. ECG coding of all study participants was carried out in a unified way sensu the Minnesota code, version of 2009^32^, by two trained specialists of the Center, with an involvement of the third expert in disputable cases. Coded ECG changes were grouped into two categories: Major ECG abnormalities and Minor ECG abnormalities. The criteria and algorithm matched the classification by Prineas RJ et al.^32^. They are presented in Table 2. As evidenced by numerous published sources, ECG changes in the presented codes (primarily Major ECG abnormalities) can indicated a presence of various cardiovascular diseases, including the likelihood of developing any cardiovascular disease, myocardial infarction, coronary artery disease, heart failure, arrhythmias and death from cardiovascular disease^1–5^.

**Table 2:** Criteria for major and minor ECG abnormalities sensu the Minnesota code classification system.

Criterion	Minnesota code
Major ECG abnormalities	
Cicatricial changes in the myocardium	1–1, 1–2
Possible cicatricial changes in the myocardium	1–3 with 4–1, 4–2, 5–1, 5–2
Isolated pronounced ST-T wave changes	4–1, 4–2, 5–1, 5–2 without 1–1, 1–2, 1–3, 3–1, 3–3, 3–2, 3–4
Left ventricular hypertrophy with pronounced ST-T wave changes	3–1 with 4–1, 4–2, 5–1, 5–2
Major rhythm and conduction abnormalities	6–1, 6–2, 6–8, 6–4-1, 6–4–2, 7–1, 7–2, 7–4, 7–8, 7–9, 8–3
Significantly prolonged ventricular repolarization	QTI > 116%
Other severe arrhythmias	8–2–1, 8–2–2, 8–4–2, (8–4–1 provided HR ≥ 140 bpm)
Minor ECG abnormalities	
Possible cicatricial changes in the myocardium	1–3 without 4–1, 4–2, 5–1, 5–2
Minor isolated ST segment and T wave changes	4–3, 4–4, 5–3, 5–4
Amplitude signs of ventricular myocardial hypertrophy	3–1, 3–2, 3–3, 3–4 without 4–1, 4–2, 5–1, 5–2
Minor conduction disorders	6–5, 6–3, 7–3, 7–6, 7–7
Minor arrhythmias	8–1–1, 8–1–2, 8–1–3, 8–1–5, 8–1–4, 8–7, 8–8, (8–4–1 provided HR < 140 bpm)
Slightly prolonged ventricular repolarization	112% ≤ QTI < 116%
Other minor ECG abnormalities	7–10, 9–1, 9–3, 9–6, 9–7

*HR* heart rate, *QTI* QT interval index, calculated as: QTI (%) = (QT/656) × (HR + 100); at QRS ≥ 120, JTI is calculated instead of QTI: JTI (%) = (JT/518) × (HR + 100), where JT = QT – QRS, and all intervals are in milliseconds (msec); *bpm* beats per minute.

### Individual covariates

Of individual variables, as covariates, we selected socioeconomic and demographic characteristics with the highest evidential level of their effect on the odd of cardiovascular disorders according to the published data sources. Gender, age, place of residence (urban vs. rural) of study subjects were identified from filled questionnaires, along with some other variables: educational level (not higher vs. higher education), marital status (has vs. does not have a family), employment status (employed vs. jobless), income level, medicine intake, dietary patterns, alcohol consumption, smoking status (never did, quit, currently smokes), diabetes mellitus, heredity (myocardial infarction in parents and or siblings: yes/no/ not aware of).

Income level was assessed indirectly by three questions characterizing the share of income spent on food, and opinions of respondents about their financial potentials and well-being as compared with other families. Each question had five response options, which were ranked from 1 pt. (the ‘poorest’ answer) to 5 pts. (the ‘richest’ answer). From sums of points, the tertiles were calculated, in accordance with the values of which the level of income was grouped into three categories: *low*, *medium*, and *high*.

Hypertension was defined as systolic blood pressure of 140 mm Hg or higher, and/or diastolic blood pressure of 90 mm Hg or higher, and/or an intake of antihypertensive medicines by the study participant within the last 2 weeks.

The presence of obesity was determined by body mass index: its values of 30.0 kg/m^2^ and above were classified as *obese*.

Diabetes mellitus was classified given the presence of at least one of the following three criteria: a history of diabetes mellitus type 1 or 2; fasting hyperglycemia (glucose level of 7.0 mmol/L or more); intake of medications to lower glucose level. Blood sampling to determine the concentration of glucose was carried out from the median cubital vein on an empty stomach, after 12 h of fasting. The glucose level was identified via glucose oxidase method on Sapphire-400 automated biochemistry analyzer (Japan) using Human GmbH kits.

Hypercholesterolemia was classified given the following: blood level of total cholesterol of 5.0 mmol/L or more; and/or use of cholesterol-lowering medications in the past 2 weeks. Total cholesterol was determined by the enzymatic method on Abbott Architect c8000 analyzer using Abbott Diagnostic kits (USA).

The presence and level of alcohol consumption was assessed according to questionnaire data, by converting the frequency, volume and type of consumed alcoholic beverages into average daily values in grams of ethanol^33^. There was a group of subjects with zero alcohol consumption. Among those who drank alcohol, the values of the 25th and 75th percentiles were calculated, according to which a grouping was performed into the following categories of alcohol consumption: *small*, *moderate* and *excessive*.

An assessment of dietary patterns was performed sensu empirical models, allowing to integrally analyze the diets of respondents according to the actual consumption frequency of food groups. A detailed description of the selection procedure via using the method of principal component analysis, along with an analysis of Russian dietary patterns (DP), was presented in our earlier publication^34^. Overall, four DP were identified: *reasonable* (dairy products, sweets and confectionery, fruits and vegetables, cereals and pasta), *salty foods* (Vienna sausages, sausages, offal, pickles and pickled products), *meat-based* (red meat, fish and seafood, poultry), and *mixed* (legumes, pickles and pickled products, fish and seafood). According to the quantitative value of individual adherence to each of four DP, the sample was grouped into four quartiles with a higher quartile characterizing a higher adherence to the particular DP.

### Regional variables

To describe regional living conditions, an integral index assessment was employed, which was previously performed using the methodology of principal component analysis^35^. In short, to identify regional indices, publicly available data from the official website of the Federal Statistics Service of Russia (www.gks.ru) for 2010–2014 were borrowed. In total, five regional indices were identified, which were quantitative indicators reflecting a negative (negative index values) or positive (positive index values) trend in a particular region. A complete definition, average, minimum and maximum values, and standard deviations of all of the regional characteristics used are presented in Supplementary Table S1.

The *Sociogeographic Index* combined 10 characteristics: (a) mean per capita consumption of vodka; (b) mean per capita consumption of wine; (c) mean per capita consumption of low alcohol drinks; (d) mean per capita consumption of brandy and brandy spirits; (e) mean annual air temperature (negative impact on the factor); (f) forested area size in the region; (g) per capita number of crimes; (h) geographical latitude of the regional center location; (i) share of dilapidated housing; (j) shares of school students studying in the morning and afternoon shifts. In general, an increase in this index value characterizes the deterioration of the social environment. In a similar way, higher values of this index imply more northerly location of the region with correspondingly worse climatic conditions.

The *Demographic Index* is formed by five characteristics: (a) natural population growth (negative impact); (b) fertility rate (negative impact); (c) total mortality rate; (d) the proportion of people of retirement age among the population; (e) mortality caused by respiratory diseases. An increase in this index values implies aggravated demographic depression in the region with depopulation and population restructuring towards the dominance of older age groups.

The *Industrial Index* encompassed eight characteristics: (a) volume of mineral resource mining; (b) energy production; (c) mortality caused by tuberculosis; (d) mortality caused by infectious diseases; (e) mortality caused by external causes; (f) the proportion of people in the region working in hazardous working conditions; (g) population numbers in the region; (h) air emissions. An increase in this index values is indicative of an increase in the regional industrial development, primarily, due to mining and energy production, with a consequent exposure of the working and retired population to unfavorable anthropogenic factors.

There are five components forming the *Mixed Index*: (a) number of workers in fish farms; (b) mean per capita volume of paid services; (c) mean per capita number of cars; (d) ratio of men to women (negative impact); (e) geographical longitude of the regional center location. The values of mixed index are the most difficult to interpret. However, it could be assumed that with its growth, the region is characterized by a favorable socioeconomic increase in the mean per capita volume of paid services and number of cars.

The *Economy Index* is formed by five characteristics: (a) per capita volume of retail trade; (b) mean per capita household consumption; (c) Gini index value; (d) mean per capita income; (e) the level of development of manufacturing industries in the region. An increase in this index values implies the growth of economic development, income, and economic inequality of the population in the region.

### Statistical data processing

We used Pearson’s chi-squared test to compare the frequencies of categorical variables in men vs. women, and Student’s *t* test to compare mean age values. The studied variables are represented by a complex two-level sample with individual and regional characteristics; therefore, to measure associations, generalized estimating equations^36^ with stable standard errors were used, taking into account the nested structure of the data (subjects in regions). Several sets of logistic models of the odd of major and minor ECG abnormalities were performed, with the calculation of the odds ratio (OR) and 95% confidence intervals (CI). Model 1 included only regional indices. In Model 2, individual socioeconomic characteristics were added to the regional indices: age, urban vs. rural residence, family status, educational level, employment status, and income category. Model 3, characterized as comprehensive, additionally included cardiovascular risk factors: hypertension, obesity, hypercholesterolemia, diabetes mellitus, smoking status, alcohol consumption, dietary pattern, and heredity risks. The preliminary analysis yielded some interactions of gender with regional indices; hence, we decided to perform all analyses separately for men and women. The critical level of statistical significance was assumed at p ≤ 0.05. All statistical procedures were performed using the SPSS software platform, version 22 (IBM, USA).

## Results

The prevalence of major and minor ECG abnormalities, along with characteristics of all variables considered as covariates in the analysis, are presented in Table 3. Major and minor ECG abnormalities were observed, respectively, in 9.4% and 45.9% of men, and 8.4% and 34.1% of women. The prevalence of all indicators (with the exception of hypertension and diabetes) differed statistically significantly between men and women.

**Table 3:** Main characteristics of analyzed indicators in men and women.

Indicator		Women, n (%)	Men, n (%)	p-value
Region	Primorsky Krai	861 (8.5)	840 (13.3)	< 0.001
	Republic of North Ossetia	1401 (13.9)	615 (9.8)	
	Volgograd region	975 (9.8)	448 (7.1)	
	Vologda region	859 (8.5)	757 (12.0)	
	Voronezh region	991 (9.8)	571 (9.1)	
	Ivanovo region	1159 (11.5)	677 (10.7)	
	Kemerovo region	911 (9.0)	686 (10.9)	
	Krasnoyarsk region	922 (9.1)	592 (9.4)	
	Tomsk region	912 (9.0)	646 (10.2)	
	Tyumen region	1104 (10.9)	473 (7.5)	
Urban residency		7832 (77.6)	5009 (79.4)	0.005
Age, years, mean ± standard deviation		47.6 ± 11.3	44.8 ± 11.8	< 0.001
Has a family		5835 (57.8)	4849 (76.9)	< 0.001
Higher education		4142 (41.0)	2728 (43.3)	0.005
Employed		7114 (70.5)	5179 (82.1)	< 0.001
Income	Low	2197 (21.8)	760 (12.1)	< 0.001
	Medium	6466 (64.1)	3901 (61.9)	
	High	1432 (14.1)	1644 (26.0)	
Hypertension		5070 (50.2)	3224 (51.1)	0.26
Obesity		3879 (38.4)	1777 (28.2)	< 0.001
Hypercholesterolemia		6667 (66.0)	3829 (60.7)	< 0.001
Diabetes		855 (8.5)	491 (7.8)	0.13
‘Reasonable’ DP	Q1	2731 (27.1)	2426 (38.5)	< 0.001
	Q2	2303 (22.8)	1662 (26.3)	
	Q3	2444 (24.2)	1239 (19.7)	
	Q4	2617 (25.9)	978 (15.5)	
‘Salty foods’ DP	Q1	2,807 (27.8)	1094 (17.4)	< 0.001
	Q2	3119 (30.9)	1906 (30.2)	
	Q3	2169 (21.5)	1500 (23.8)	
	Q4	2000 (19.8)	1805 (28.6)	
‘Meat-based’ DP	Q1	2661 (26.4)	1185 (18.8)	< 0.001
	Q2	2267 (22.4)	1438 (22.8)	
	Q3	3066 (30.4)	1971 (31.3)	
	Q4	2101 (20.8)	1711 (27.1)	
‘Mixed’ DP	Q1	2704 (26.8)	1890 (30.0)	< 0.001
	Q2	2698 (26.7)	1352 (21.4)	
	Q3	2291 (22.7)	1379 (21.9)	
	Q4	2402 (23.8)	1684 (26.7)	
Alcohol consumption	None	2305 (22.8)	998 (15.8)	< 0.001
	Small	3709 (36.7)	621 (9.8)	
	Moderate	3328 (33.0)	2079 (33.0)	
	Excessive	753 (7.5)	2607 (41.4)	
Smoking	None	7879 (78.0)	2055 (32.6)	< 0.001
	Quit	1027 (10.2)	1821 (28.9)	
	Smokes	1189 (11.8)	2429 (38.5)	
Myocardial infarction in close relatives	No	7251 (71.8)	4511 (71.6)	< 0.001
	Yes	1864 (18.5)	998 (15.8)	
	Not aware of	980 (9.7)	796 (12.6)	
Major ECG abnormalities		847 (8.4)	595 (9.4)	0.021
Minor ECG abnormalities		3438 (34.1)	2893 (45.9)	< 0.001

*DP* dietary pattern.

Without taking into account individual characteristics, the odd of major ECG abnormalities in men declined with the growth of both Sociogeographic Index and Mixed Index, and increased with the growth of both Demographic Index and Industrial Index (Table 4). The addition of age and individual socioeconomic characteristics to the model led to a reduction in association with Sociogeographic Index. Further addition of individual cardiovascular risk factors to the model did not modify the association. In the comprehensive model, the odd of major ECG abnormalities was higher in demographically depressed regions (1.08: 1.04–1.12), industrialized regions (1.12: 1.07–1.17), but was lower with increasing Mixed Index (0.97: 0.95–0.98).

**Table 4:** Odd of major ECG abnormalities vs. regional indices.

Regional indices	Model 1		Model 2		Model 3	
	OR	95% CI	OR	95% CI	OR	95% CI
Men						
Sociogeographic	0.91*	0.85–0.98	0.96	0.89–1.03	0.97	0.89–1.06
Demographic	1.04*	1.01–1.08	1.09*	1.05–1.13	1.08*	1.04–1.12
Industrial	1.17*	1.12–1.22	1.16*	1.11–1.21	1.12*	1.07–1.17
Mixed	0.96*	0.95–0.97	0.96*	0.95–0.97	0.97*	0.95–0.98
Economy	1.00	0.95–1.06	0.97	0.93–1.02	0.96	0.90–1.02
Women						
Sociogeographic	0.89*	0.80–0.98	0.91*	0.84–0.99	0.92	0.85–1.00
Demographic	1.01	0.95–1.08	1.01	0.95–1.06	1.00	0.95–1.06
Industrial	1.06	0.96–1.17	1.11*	1.07–1.16	1.12*	1.07–1.16
Mixed	0.89*	0.79–0.99	0.94	0.86–1.04	0.95	0.86–1.04
Economy	1.03	0.91–1.17	0.95	0.91–1.00	0.94*	0.89–0.99

Model 1 contains all regional indexes; Model 2, in addition to regional indices, includes individual socioeconomic characteristics; Model 3, besides regional and socioeconomic characteristics, comprises cardiovascular risk factors; *p ≤ 0.05.

In women, the odd of major ECG abnormalities in Model 1 was inversely associated with both Sociogeographic Index and Mixed Index. The addition of age and individual socioeconomic characteristics (Model 2), and of individual cardiovascular risk factors (Model 3) to the base model led to a reduction in association with these indices. However, new associations were identified in the comprehensive model (Model 3): an increase in the industrial development of the region was associated with an increase in the odd of major ECG abnormalities (1.12: 1.07–1.16); whereas an increase in the economic development of the region was associated with a decline in the odd of major ECG abnormalities (0.94: 0.89–0.99).

The odd of minor ECG abnormalities in men in all three models was inversely associated with both Mixed Index and Economy Index (Table 5). In the comprehensive model, OR = 0.95: 0.93–0.98; and OR = 0.86: 0.80–0.93, respectively. In women, in models that took into account solely regional indices (Model 1), associations were also identified for both Mixed Index and Economy Index. However, when individual characteristics were added to the regression model, just the inverse association of the Economy Index remained statistically significant (0.92: 0.87–0.99).

**Table 5:** Odd of minor ECG abnormalities vs. regional indices.

Regional indices	Model 1		Model 2		Model 3	
	OR	95% CI	OR	95% CI	OR	95% CI
Men						
Sociogeographic	0.99	0.89–1.10	1.00	0.89–1.11	0.99	0.88–1.10
Demographic	0.98	0.91–1.05	0.97	0.91–1.04	0.97	0.91–1.04
Industrial	1.01	0.94–1.09	1.01	0.94–1.09	1.00	0.92–1.08
Mixed	0.95*	0.92–0.97	0.95*	0.93–0.98	0.95*	0.93–0.98
Economy	0.87*	0.80–0.94	0.87*	0.81–0.92	0.86*	0.80–0.93
Women						
Sociogeographic	0.96	0.91–1.02	0.96	0.91–1.02	0.97	0.91–1.04
Demographic	1.03	0.99–1.07	1.03	0.99–1.06	1.03	0.98–1.07
Industrial	1.01	0.97–1.05	1.01	0.96–1.07	1.01	0.96–1.06
Mixed	0.96*	0.93–0.99	0.98	0.95–1.01	0.97	0.94–1.01
Economy	0.94*	0.89–0.98	0.92*	0.86–0.98	0.92*	0.87–0.99

Model 1 contains all regional indexes; Model 2, in addition to regional indices, includes individual socioeconomic characteristics; Model 3, besides regional and socioeconomic characteristics, comprises cardiovascular risk factors; *p ≤ 0.05.

## Discussion

The results of our study demonstrated that even when taking into account individual socioeconomic characteristics, as well as behavioral and clinical factors of cardiovascular risk, regional living conditions were associated with major and minor ECG abnormalities. An increase in the odd of ECG abnormalities was noted with an increase in demographic depression and industrial development of regions (Demographic Index and Industrial Index), as well as with deterioration of the economic living conditions of the population (Economy Index and Mixed Index). Common for men and women were the direct influence of the regional industrial development on the odd of major ECG abnormalities and the reverse effect of the regional economic development on minor ECG abnormalities. Besides, only men were characterized by a direct association of the demographic depression of regions with major ECG abnormalities, as well as by inverse associations of the Mixed Index with major and minor ECG abnormalities. In women, unlike men, regional economic development was associated with both minor and major ECG abnormalities.

The increase in the odd of major ECG abnormalities with the growth of regional industrial development could be associated with the direct impact of technogenic pollution on both working and retired population. Besides, the Industrial Index includes indicators directly implying the exposure of the population to anthropogenic pollution: the proportion of people in the region working in hazardous working conditions and air emissions. Numerous published data confirmed the effect of ambient air pollution on cardiovascular morbidity and mortality^37,38^; the latter is also characteristic of industrial pollution^39,40^. Previously, on the same sample as in this study, we revealed that the growth of the regional industrial development was associated with an increase in the odd of developing myocardial infarction and overall cardiovascular risk in a 5-year prospective follow-up^41^. Therefore, the obtained results exhibited negative impact of anthropogenic pollution on the myocardium, which manifested itself to a greater extent in severe (major) ECG abnormalities.

An impact of the ‘*economic environment’* (Economy Index and, partially, Mixed Index) in this study was positive and overall matched similar foreign data^23,25,26,28,42^. However, a number of studies indicated an adverse effect of income inequality (for example, an increase in the Gini index) on the risk of CAD^29^. In our study, the growth of income inequality in the population was complexly related to the economic development of the region. Therefore, it was not possible to isolate its impact on the odd of ECG abnormalities from related factors. It is quite possible that our approach to a comprehensive evaluation of living conditions more adequately assesses the impact of the complex interdependence of regional economic characteristics.

An increase in the odd of major ECG abnormalities revealed in men in demographically depressed regions was consistent with previously obtained data on the increase in cardiovascular risk over a 3-year follow-up period^41^. As in the prospective study, an impact of the Demographic Index was less pronounced (compared with the Industrial Index and Mixed Index), which could be due to indirect mediation by other spatial characteristics. After all, as a rule, regional demographic depression consists of a combination of other causes: e.g. the decline of industrial production and deterioration of economic and social conditions. That is, in fact, the demographic depression of the region is a derivative of other territorial features of living conditions. Based on the obtained results, it can be assumed that in this case an influence of primary causes was more pronounced than the effect of combined consequences.

Noteworthy are the gender differences in the associations of living conditions with major and minor ECG abnormalities, which, apparently, were caused by more active social role of men. Men, as the head of the household in Russian society, are responsible for well-being and prosperity, which, accordingly, can be reflected in the form of stress-induced deterioration in their health under adverse living conditions. For example, it has been shown that men were more likely to suffer from more mental health problems than women when faced with situations of high wealth inequality^43^. At the same time, although higher income inequality at the level of large regions determines higher chances of depression at the individual level in both women and men, such associations are more pronounced in the latter^44^. However, it should be noted that studies on the impact of living conditions on the odd of CAD revealed multidirectional patterns. In some studies, associations were stronger among men^25^, while in other studies, they were more pronounced in women^27^, or else, similar between genders^26,29^.

Judging from available publications, our study was the first to characterize associations of regional living conditions with ECG abnormalities. From a practical standpoint, our data constitute the first attempt to comprehend why there are such significant differences in cardiovascular risk among regions of the Russian Federation. The indisputable advantage of our study is the quality of its design, specifically, the correct setting of tasks and proper collection of epidemiological data, large total sample size and the number of regional samples, and the use of adequate methods of statistical analysis. Among the shortcomings of our study, we should mention its cross-sectional design, limiting the analysis in terms of causality of identified patterns. However, the studied socioeconomic and demographic characteristics have the highest evidential level of their effect on the likelihood of cardiovascular disorders, and OR values may indicate the true influence of the studied characteristics on the occurrence of ECG abnormalities.

## Conclusion

The study demonstrated an effect of regional living conditions of the Russian population on the odd of major and minor ECG abnormalities. The most stable and logically explainable relationships were obtained for industrial and economic characteristics of living conditions, which confirmed the adverse impact of environmental and economic factors on cardiovascular risks of the population. From a practical standpoint, our results provided evidence-based data confirming the need to take into account regional differences in the living conditions of people for predicting, planning and implementing health care programs aimed at improving population health.

## Supplementary Information

Supplementary Table S1.

## Data Availability

Study data are available upon reasonable request from the authors.
