# Supplementary material for: Major and minor ECG abnormalities depending on regional living conditions in Russia
Source: Sci Rep. 2023 Jun 1;13:8934. doi: 10.1038/s41598-023-35947-2 (PMC10235056; doi:10.1038/s41598-023-35947-2)
Supplement: Supplementary file 1 — Supplementary Table S1. [file 41598_2023_35947_MOESM1_ESM.docx]

**Supplementary Table S1.** Baseline characteristics of the regional characteristics, Epidemiology of Cardiovascular Diseases in the Regions of the Russian Federation (ESSE-RF), Russian Federation, 2013–2014

| Characteristics | Mean | Standard Deviation | Minimum | Maximum |
| --- | --- | --- | --- | --- |
| **Sociogeographic Index** | | | | |
| Sales of vodka, liqueurs, and spirits*, liters per capita | 9.0 | 3.2 | 2.6 | 13.4 |
| Sales of brandy and brandy spirits*, liters per capita | 0.64 | 0.23 | 0.24 | 1.12 |
| Sales of low-alcohol beverages, with alcohol content of under 9%, excluding wines, champagne, and beer*, liters per capita | 1.94 | 1.46 | 0.12 | 6.00 |
| Sales of champagne and sparkling wines*, liters per capita | 1.59 | 0.50 | 0.54 | 2.48 |
| Average annual temperature, degrees Celsius | 4.6 | 3.1 | 0.9 | 10.4 |
| Timberland area*, % | 38.1 | 26.4 | 4.3 | 77.3 |
| Number of recorded crimes*, per 100,000 people | 1854 | 513 | 902 | 2525 |
| Location of the regional center, north latitude, degrees | 52 | 5 | 43 | 59 |
| Decrepit and dilapidated housing in the total floor area of all the available housing*, % | 3.05 | 1.31 | 0.98 | 5.20 |
| Proportion of students of state and municipal general education institutions (excluding evening schools), second and third shifts, at the beginning of the academic year*, % of the total number of students | 14.7 | 5.3 | 9.3 | 23.6 |
| **Demographic Index** | | | | |
| Natural increase rate*, per 1000 people | -0.85 | 3.30 | -6.14 | 4.40 |
| Crude birth rate*, number of newborns per 1000 people | 13.1 | 1.8 | 10.6 | 16.5 |
| Crude mortality rate*, number of deceased per 1000 people | 13.8 | 2.0 | 10.5 | 17.0 |
| Population of unemployable age at the year-end*, % | 23.1 | 2.4 | 19.7 | 26.9 |
| Mortality rate from diseases of the respiratory system*, per 100,000 people | 59.0 | 17.0 | 22.7 | 93.5 |
| **Industrial Index** | | | | |
| Volume of shipped own-produced goods, in-house works, and services related to mineral extraction*, million rubles | 123 085 | 150 572 | 407 | 451 893 |
| Volume of shipped own-produced goods, in-house works, and services related to electric power, gas, and water production and distribution*, million rubles | 55 886 | 32 474 | 6633 | 108 173 |
| Mortality rate from infections and infestations*, per 100,000 people | 26.8 | 13.5 | 10.3 | 49.7 |
| Mortality rate from tuberculosis*, per 100,000 people | 14.3 | 7.7 | 6.2 | 29.0 |
| Mortality rate from external causes*, per 100,000 people | 150.1 | 35.8 | 68.1 | 214.0 |
| Proportion of people employed in toxic and/or hazardous jobs—in the mineral extraction, manufacturing, electric power, gas, and water production and distribution, construction, transport and communication sectors—of the total workforce, at the year-end of 2014, for all forms of incorporations, % | 43.0 | 12.0 | 22.6 | 66.8 |
| Population size at the year-end*, thousand people | 1923.0 | 831.0 | 707.4 | 3213.2 |
| Emissions of pollutants from stationary sources into the atmosphere*, 1000 tons | 512.9 | 724.6 | 4.4 | 2 488.8 |
| **Mixed Index** | | | | |
| Per capita amount of paid services*, in rubles | 36 326 | 8 139 | 27 882 | 57 789 |
| Number of private passenger cars at the year-end*, per 1000 people | 258 | 36 | 204 | 336 |
| Male/female ratio at the year-end*, women per 1000 men | 1160 | 37 | 1085 | 1232 |
| Location of the regional center, east longitude, degrees | 64 | 28 | 39 | 131 |
| **Economy Index** | | | | |
| Per capita retail turnover*, at effective prices, in rubles | 122 696 | 26 919 | 98 422 | 181 820 |
| Per capita actual final consumption of households **, in current market prices, in rubles | 181 712 | 26 128 | 147 439 | 223 548 |
| Gini Index* | 0.39 | 0.02 | 0.36 | 0.44 |
| Per capita income per month*, in rubles | 19 106 | 2 817 | 15 735 | 23 920 |
| Volume of shipped own-produced goods, in-house works, and services related to manufacturing*, million rubles | 336 423 | 239 376 | 15 658 | 721 966 |

* average for 2010–2014; ** average for 2010–2013.
